# 5-MeO-DMT induces sleep-like LFP spectral signatures in the hippocampus and prefrontal cortex of awake rats

**DOI:** 10.1038/s41598-024-61474-9

**Published:** 2024-05-17

**Authors:** Annie C. Souza, Bryan C. Souza, Arthur França, Marzieh Moradi, Nicholy C. Souza, Katarina E. Leão, Adriano B. L. Tort, Richardson N. Leão, Vítor Lopes-dos-Santos, Sidarta Ribeiro

**Affiliations:** 1 Brain Institute, Federal University of Rio Grande do Norte, Natal, Brazil; 2 Department of Psychology, Florida State University, Tallahassee, USA; 3 Donders Institute for Brain, Cognition and Behaviour, Radboud University, Nijmegen, The Netherlands; 4 Netherlands Institute for Neuroscience, Amsterdam, The Netherlands; 5 Department of Neuroscience and Behavioural Sciences, School of Medicine, University of São Paulo, Ribeirão Preto, Brazil; 6 Medical Research Council Brain Network Dynamics Unit, Nuffield Department of Clinical Neurosciences, University of Oxford, Oxford, UK; 7 Center for Strategic Studies, Oswaldo Cruz Foundation (FIOCRUZ), Rio de Janeiro, Brazil

**Keywords:** 5-MeO-DMT, Psychedelics, Hippocampal oscillations, Prefrontal cortex, Sleep, Neurophysiology

## Abstract

5-methoxy-N,N-dimethyltryptamine (5-MeO-DMT) is a potent classical psychedelic known to induce changes in locomotion, behaviour, and sleep in rodents. However, there is limited knowledge regarding its acute neurophysiological effects. Local field potentials (LFPs) are commonly used as a proxy for neural activity, but previous studies investigating psychedelics have been hindered by confounding effects of behavioural changes and anaesthesia, which alter these signals. To address this gap, we investigated acute LFP changes in the hippocampus (HP) and medial prefrontal cortex (mPFC) of freely behaving rats, following 5-MeO-DMT administration. 5-MeO-DMT led to an increase of delta power and a decrease of theta power in the HP LFPs, which could not be accounted for by changes in locomotion. Furthermore, we observed a dose-dependent reduction in slow (20–50 Hz) and mid (50–100 Hz) gamma power, as well as in theta phase modulation, even after controlling for the effects of speed and theta power. State map analysis of the spectral profile of waking behaviour induced by 5-MeO-DMT revealed similarities to electrophysiological states observed during slow-wave sleep (SWS) and rapid-eye-movement (REM) sleep. Our findings suggest that the psychoactive effects of classical psychedelics are associated with the integration of waking behaviours with sleep-like spectral patterns in LFPs.

## Introduction

Classical psychedelics such as 5-methoxy-N,N-dimethyltryptamine (5-MeO-DMT) comprise structural analogues of serotonin that in humans induce major changes in perception, movement, emotion and cognition^1–11^. In rodents, the acute behavioural effects of classical psychedelics include changes in locomotion, space occupancy, and stereotyped behaviours such as *wet dog* shaking, head twitching, tremors, and backward gaiting^12,13^. Rats under acute effects of d-LSD, another serotonin analogue, decreased the number of arms entries in the Y-maze task^14^, and presented inhibition of fighting in a shock-elicited fighting paradigm when treated with high doses of d-LSD or 5-MeO-DMT^15^. Head-twitching increases in a dose-dependent manner, while mating ultrasonic vocalizations are suppressed by 5-MeO-DMT^16^. The startle response is altered upon psychedelic dosing in rodents^17^. Furthermore, a conditioned avoidance response was disrupted progressively over time after NN-DMT dosing, with a peak effect at 8 min post-dosing^18^.

More recently, the electrophysiological dynamics that underlie acute psychedelic experiences have begun to be investigated in rodents. The spike rates of mPFC neurons recorded from anaesthetised rats have been shown to increase after dosing of the classical psychedelic DOI^19^, but another study found that mean firing rates either increased or decreased in different mPFC neurons after DOI dosing, while paired cell synchrony did not change and LFP signals showed decreased power in the gamma frequency band^20^. DOI also led to a decrease in prefrontal cortical slow oscillations in anaesthetised animals, possibly decreasing global synchronisation^21^. Likewise, the intravenous dosing of 5-MeO-DMT to anaesthetised rats led to either decreased or increased firing rates in cortical pyramidal neurons, with a corresponding reduction in the power of slow oscillations recorded from the mPFC. Another study showed that 5-MeO-DMT alters the cortico-thalamic activity in freely behaving mice, leading to increased delta power in the V1 cortex, increased beta power in the medial mPFC, decreased beta power in the medial thalamus, and increased gamma power in the medial mPFC, as well as increased coherence in the beta frequency band across the V1 cortex, the medial thalamus and the medial mPFC; and increased coherence in the theta frequency band between the medial mPFC and the medial thalamus^22^.

Interpreting the electrophysiological findings is complicated by the behavioural confounders such as sleep, anaesthesia, and speed, and by the neuroanatomical constraint of exclusively evaluating cortical signals.

Classical psychedelics, like psilocin, have been shown to impact sleep maintenance and cortical oscillations in mice^23^, while the mental states they induce in humans share similarities with dreaming^24–27^. This intriguing paradox remains unresolved. Most relevant studies were conducted on anesthetised animals, which prevents the manifestation of both waking and physiological sleep. In freely moving mice, psilocin delayed REM sleep onset and decreased NREM sleep maintenance, resulting in heightened 4 Hz oscillations^23^. An initial report found that 5-MeO-DMT reduced power in the theta frequency range (5–10 Hz) and increased slow wave activity while animals remained awake, suggesting a hybrid state with features of both waking and slow-wave sleep^28^. However, this notion is limited by its reliance on cortical data uncontrolled for speed. To date, no electrophysiological studies of psychedelics have controlled for speed or investigated beyond the cerebral cortex^21–23,28^.

The hippocampus (HP) plays a key role in rodent navigation and cognition, and its neural activity is characterised by various rhythms. In particular, theta oscillations dominate hippocampal activity during active behaviour and REM sleep^29^. Further, theta amplitude is positively correlated with animal speed during waking periods^30,31^ and strongly influences cortical signals^32^. The theta cycle is thought to organise multiple operations in the hippocampal circuit, such as the activation of different pathways^33,34^. Additionally, theta-nested gamma oscillations are thought to reflect different circuits within the hippocampal formation^35–37^. Notably, while both active waking and REM sleep exhibit pronounced theta oscillations, gamma-range activity is more robust during waking than in REM sleep^38^. Throughout non-REM sleep, the hippocampus is predominantly governed by large irregular activity^39,40^, manifested through intermittent patterns such as dentate spikes and sharp-wave ripples ^41–43^. These patterns predominantly emerge in spectral analysis within the delta range (< 4 Hz).

In the absence of data from both the HP and cortex, and without controlling for speed and behavioural variability, it is not possible to draw any conclusions from rodents about the sleep-waking spectral features of LFP signals during acute psychedelic states. To fill this gap, we combined chronic simultaneous LFP recordings from the HP and mPFC with a detailed quantitative analysis of speed as well as stereotyped behaviours after dosing of 5-MeO-DMT. Based on the current literature, we hypothesised that the main brain oscillations should all be altered during the acute psychedelic state. Also, we hypothesised that 5-MeO-DMT would induce an atypical waking state that may spectrally resemble sleep states.

## Methods

### Animals

A total of 17 adult male rats were used (*Rattus norvegicus*, Wistar, 250–350 g, ~ 2 months old). The animals were housed in an appropriate vivarium under controlled temperature and humidity, with lights on at 6:00 and lights off at 18:00. All methods were performed in accordance with the ARRIVAL guidelines and relevant regulations, including approval by the Ethics Committee at UFRN (permit CEUA #11/2015) and by the Brazilian Health Regulatory Agency (ANVISA AEP # 018/2021).

### Drug

The study used 5-MeO-DMT (Merck) dissolved in DMSO (0.27mg/ul), and then diluted in 0.9% saline to final doses.

### Electrode manufacturing

Electrodes were designed and handmade as described elsewhere^44,45^. The electrode arrays were designed to be implanted in the mPFC (16 channels) and the hippocampus (16 channels) according to Paxinos Atlas^46^. In each area, two rows of 8 electrodes were implanted along the anteroposterior axis, with 250 µm of inter-electrode space.

### Surgery and post-operative animal care

Experimental animals were subjected to surgery for electrode implantation as described in Souza et al.^45^. Briefly, anaesthesia was induced with inhaled isoflurane at 5% followed by intraperitoneal ketamine and xylazine at respective doses of 100 mg/kg and 8 mg/kg. When needed supplemental doses of ketamine and xylazine were applied to maintain anaesthesia. Electrodes and the cannula were then implanted aiming at the dorsal hippocampus (Anteroposterior (AP): − 3.28 mm; Mediolateral (ML): 2.0 mm; Dorsoventral (DV): 2.2 mm); the prefrontal cortex (AP: + 3.72 mm; ML: − 0.5 mm; and DV: 3.5 mm); and the third ventricle (AP: − 0.96 mm; ML: 1.8 mm; and DV: 3.2 mm). Following the surgery animals were treated with analgesic, anti-inflammatory, and antibiotic drugs and given time to recover.

### Experimental design

**Figure 1:**
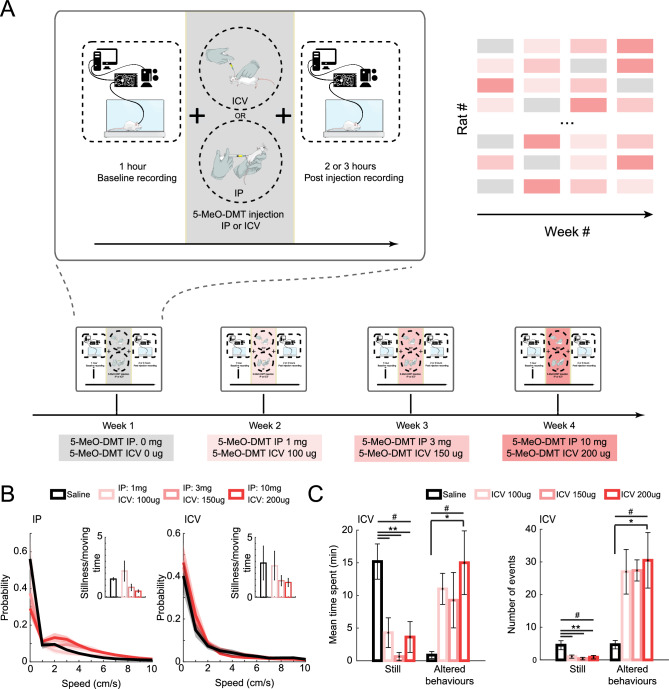
Experimental design and behavioural results. (**A**) Animals were submitted to 4 weeks of experiments for 5-MeO-DMT. On each experimental session we recorded 1 h of baseline followed by 2 or 3 h after drug injection; Two additional 1-h recordings were made one day before and after the experiment but are not included in this work. Each week we treated animals with a different dose or vehicle (saline), in a randomised order among animals. (**B**) Normalised histogram of animal speed after IP (left) and ICV (right) injection of saline or different doses of 5-MeO-DMT. Insets show the average ratio between stillness (< 1 cm/s) and moving (between 1 and 8 cm/s). Error bars and shaded areas denote SEM. (**C**) Mean time spent (left panel) and the number of events (right panel) for the ‘Still’ behaviour and ‘Altered behaviours’ (see Supp Fig. 1). ANOVA #*p* < 0.05, (‘Still’: Mean time: F(3,20) = 7.6747, Number of events: F(3,20) = 5.3164. ‘Altered behaviours’: Mean time spent: F(3,20) = 3.2362, Number of events: F(3,20) = 3.8690), with post hoc test *p < 0.05; **p < 0.01.

Animals were submitted to four, weekly-spaced experimental sessions in random order (Fig. 1A; 1 to 8 sessions per rat, totalling 54 drug and 20 saline sessions). Each experimental session consisted of three consecutive days that started with a one-hour recording of the animal in the arena (55.5 × 65 × 45 cm). On the second day, after the baseline recording, animals were injected with one of the three 5-MeO-DMT doses (for ICV groups: 100 ug, 150 ug, or 200 ug diluted in 2 ul of saline; for IP groups: 1 mg, 3 mg, or 10 mg diluted in 0.3 ml of saline) or saline (negative controls, referred to as 0 g ICV or IP) and recorded for an additional two to three hours. Electrophysiology and animal behaviour were recorded at each session. All experiments reported here refer to the second day of each experimental week. The experimental unit was the data obtained from each animal in each experimental session; Supp Table S1 specifies the number of experimental units allocated to each group. We selected a moderate sample size because the dose-dependent effects of 5-MeO-DMT in the HP and mPFC of freely behaving animals were evaluated for the first time in the present study, and therefore, the initial intention was to gather basic evidence regarding the dose-dependent effects of 5-MeO-DMT in more complex experimental designs. The allocation of experimental units to control and treatment groups was randomized using a computer based random order generator. ACS was aware of the group allocation during the allocation, the conduct of the experiment, and the outcome assessment; BCS was aware of group allocation during the data analysis. A few experimental units had problems with either the video of the behavior or the recorded LFPs. In those cases, the unit was analyzed only when appropriate. Experimental units in which the ICV injection showed any signs of leaking or injection resistance (due to cannula clogging) were completely excluded from all analyses. A few experimental units had problems with either the video of the behavior or the recorded LFPs. In those specific cases, the unit was only used in analysis that did not require the missing data (e.g., behavioral analysis when the electrophysiological recordings were missing). Moreover, experimental sessions in which the animal mostly slept after saline injection were excluded from the speed-controlled analyses due to lack of moving data points. For the slow and mid gamma analyses, only recording units that had at least one electrode containing the respective gamma were included. Identification of the slow or mid gamma channels was done by blind inspection of the average spectral decomposition triggered by theta peaks. Those exclusion criteria were defined a priori.

Before the first experimental session, animals were handled (~ 20 min) and habituated to the experimental room and box for at least three days (1 h per day). Experimental room temperature of 24 °C was constant for all sessions. Experiments were done in the afternoon or beginning of the evening (14–18 h) under dim light. For intracerebroventricular dosing, we used a Hamilton syringe of 10 ml connected to a polyethylene tube and a thinner cannula to fit in the implanted cannula. The volume dosing of 1ul unilaterally was manually administered for a duration of 30 s approximately.

### Recordings

The electrophysiological and video recordings were conducted using the Plexon Omniplex System or Intan Technologies coupled to a Logitech C920 webcam (30 Hz). Plexon and Intan signals were recorded at 40 kHz and 30 kHz, respectively, and down sampled to 1 kHz for LFPs. Intan recordings were synchronised to video using a TTL pulse in a pseudorandom frequency to the Intan and a LED was placed in the filming area of the camera. No video alignment pre-processing was done for Plexon data since it is automatically synchronised.

### Behavioural analyses

The animals were tracked using Cineplex from Plexon or IdTracker to detect the animals’ centre of mass. Once the tracking was performed, the animal’s speed was calculated. Further behavioural analyses were done using Cineplex Editor.

### LFP analyses

All the LFP analyses described below were performed in Matlab. Behavioural and electrophysiological changes during sleep were measured using ANOVA test followed by a Tukey–Kramer post-hoc test when applicable. Normality was tested using a Kolmogorov–Smirnov test. For speed (and power) controlled analyses custom subsampling procedures and generalized linear models (GLM) were used. The level of significance was set either at *p* < 0.05, *p* < 0.01 or *p* < 0.001.

### Power spectrum density estimates

We analysed the time-varying frequency content of the LFP signal using a spectrogram, to calculate the following outcome parameters: delta (0.5–4 Hz), theta (5–12 Hz), low gamma (25–50 Hz), mid gamma (50–100 Hz), spectral state maps, mean duration of each wake-sleep state, and transition probabilities across states. The signal was divided into 1-s overlapping segments, with a 50% overlap. The power spectral density (PSD) for each segment was calculated using Welch’s method, applying a Hamming window. The analysis used a sampling frequency of 1000 Hz and 4096 FFT points.

### Speed matching analysis

**Figure 2:**
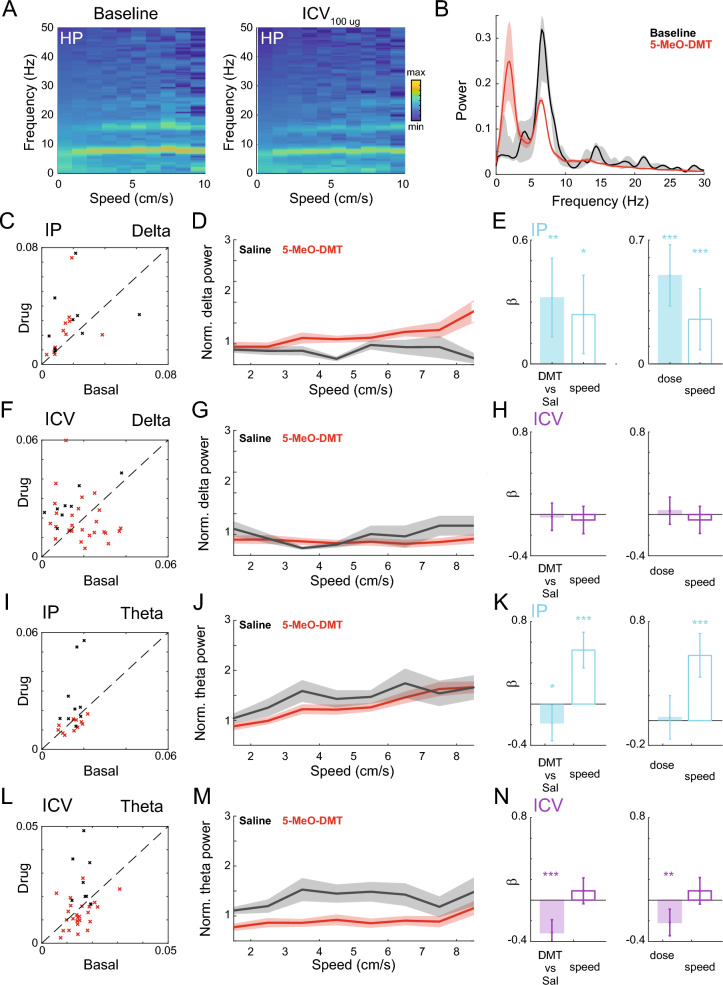
Changes in hippocampal delta and theta power after 5-MeO-DMT dosing. (**A**) Mean power spectrum at different bins of speed for a 30 min block of baseline (left) and 5-MeO-DMT at 100 ug (right). (**B**) PSDs of one session obtained using the bootstrap procedure to equalise the speeds of drug and baseline periods (see methods). The shaded area represents the 95% confidence interval. (**C**) Mean delta power acquired from the bootstrap analysis during baseline and after IP dosing. Each cross corresponds to one experimental session after dosing (5-MeO-DMT: red; Saline: grey). (**D**) Mean normalised delta power of saline and drug conditions for each speed bin. (**E**) Coefficients of GML models estimated using the normalised power in D accounting for (left) animal speed and drug (5-MeO-DMT or saline), and (right) animal speed and normalised dose (0%: saline; 100%: maximum dose). (**F**–**H**) As in C–E, but for ICV injections. (**I**) Mean theta power acquired from the bootstrap analysis during baseline and after IP dosing. Each cross corresponds to one experimental session during the first 30 min after dosing (5-MeO-DMT: red; Saline: grey). (**J**) Mean normalised theta power of saline and drug conditions for each speed bin. (**K**) Coefficients of GML models estimated using the normalised power in D accounting for (left) animal speed and drug (5-MeO-DMT or saline), and (right) animal speed and normalised dose (0%: saline; 100%: maximum dose). (**L–N**) Similar to I–K, for ICV injections.

To address any potential behavioural differences between conditions, we employed a bootstrap procedure^47^ to match the speed distributions of animals between conditions (baseline and post-injection sessions). Specifically, we randomly sampled half of the windows in a 30 min period post-injection and paired them with baseline windows based on the speed of the animals, thereby ensuring equivalent speed distributions across sessions. This procedure was repeated 500 times for each recording day. We then compared the mean delta and theta power of baseline and post-injection (e.g. Fig. 2C, F, I, L) conditions.

#### GLM for assessing drug effect on delta and theta

To further investigate the effects of the drug on the power of delta and theta, we used a generalised linear model (GLM) approach. For each recording day, we computed the mean delta or theta power for different speed bins (1 cm/s wide bins with minimum values ranging from 1 to 8 cm/s). These power values were then divided by the mean obtained for the first speed bin (1–2 cm/s) in their corresponding baseline session. We used a linear regression model to predict the power values based on both the speed values and a categorical variable indicating whether the data were obtained from a 5-MeO-DMT or saline session. This approach allowed us to assess if the injection of 5-MeO-DMT was significantly different from saline administration after accounting for differences in speed. To assess for a potential dose–response relationship, we modified the model by replacing the binary categorical variable (5-MeO-DMT or saline) with the actual dose of the drug (with saline assigned a dose of 0). In all those analyses the channel with higher theta/delta ratio was used for both brain regions. The same analysis was done individually for both IP and ICV administrations.

#### GLM for assessing drug effect on gamma oscillations

To investigate theta-nested gamma oscillations, we ran a matching analysis to determine whether any effects on gamma oscillations were in addition to what would be expected from theta power reductions and speed changes. To achieve this, we randomly selected theta cycles from the post-injection period and paired them with cycles from the baseline period (without repetition) that had similar speed and theta power. We defined a pair of cycles as “similar” when the difference between their speed and theta power values was less than 5% of the difference between the 10th and 90th percentiles of their overall distribution.

After matching the speed and theta distributions, we calculated the average gamma power of the paired theta cycles for each session. Subsequently, we normalised the average gamma power value obtained from the post-injection session by the mean value obtained from the baseline session (difference between the means divided by their sum). Finally, we fit a GLM as before, to predict relative gamma power from the speed and from categorical variables indicating whether each observation was obtained from a 5-MeO-DMT or saline session. Notice that this essentially is equivalent to a t-test but after matching the potential confounding variables (speed and theta power). Further, the same analysis was run but replacing this categorical variable by a specific dose (with saline assigned a dose of 0) to test for a dose–response relationship. These analyses were done individually for both IP and ICV administrations, and for slow and mid gamma oscillations.

To investigate the coupling between gamma amplitude and theta phase, we calculated theta-peak triggered averages of the (slow or mid) gamma envelope. Inconsistent relationships between gamma power and theta phase would result in flat averages, while consistent relationships would produce prominent theta components in these averages. Thus, quantify the strength of the coupling by calculating the power within the theta range of these theta-peak triggered averages. The theta cycles used for this analysis were matched for speed and theta power, as in the previous analysis. To assess the effects of 5-MeO-DMT on this modulation, we used similar GLMs as the ones used to assess the effect on gamma power.

#### Filtering theta and gamma oscillations

To extract the theta component, we filtered the LFP signals using a FIR filter for the band 5–12 Hz. To define the order of the filter we used the *eegfilt* function of the EEGLAB toolbox^48^. Theta peaks were detected as local maxima of the filtered signals. To extract the envelopes of gamma oscillations, we first applied a similar FIR filter to each LFP signal with the corresponding gamma frequency band (slow: 25–50 Hz, mid: 50–100 Hz). We then estimated the envelope of each gamma by applying the Hilbert transform to the filtered signal.

#### Wake-sleep cycle state maps

To study the wake and sleep states in the baseline, we used the spectral state map analysis described in Gervasoni et al.^38^ combined with the animal tracking obtained from the video recordings and data-driven thresholds of electrophysiological markers. Briefly, we first computed a spectrogram for each channel using a 1-s-window with 0.5 s of overlap. Then, we calculated two frequency ratios corresponding to delta and theta relative power; and gamma relative power (F < 4.5 Hz over F ≤ 9 Hz; and F < 20 Hz over F < 55 Hz). For each ratio, the first principal component was computed (across channels) and then used to plot the state map for the baseline of each recording session. From the video tracking (obtained from Cineplex or IdTracker, see above), we computed the mean animal velocity in the same timescale and periods of movement (> 1 cm/s) were then used as landmarks to the identification of the wake stage in the state map. The region of each state (WK, SWS and REM) on the state map was then manually identified. For that, we also used the rest of the session to account for the possibility of not having sleep during the baseline, but always having the baseline period as a reference. Notice that the ‘canonical’ sleep staging can only be done for baseline periods, since the drug conditions may induce an altered state per se, that may not fit in the same classifications. The classification of REM-, SWS- and WK-like stages after the drug dosing was performed to characterise these altered states. However, it could be behaviourally attested that animals were, in general, moving after the first 15 min of drug dosing (i.e., were in a WK state).

Finally, we computed the mean duration of each wake-sleep state for saline and drug conditions, as well as the mean velocity of each state (across pooled episodes in Fig. 6 and Supp Fig. S2). We also computed the transition probabilities from going from each of the states to the others. For that analysis, unclassified periods with less than 20 s occurring between the same state were included in that state.

## Results

### Behavioural effects of 5-MeO-DMT

First, we quantified the behaviours after 5-MeO-DMT ICV dosing (Fig. 1B,C; Supp Fig. S1). Besides some stereotyped behaviours already mentioned in the literature (e.g., *wetdog* shake), we quantified abnormal behaviours through empirical observation. More specifically, we show here the 10 behaviours most observed across animals: intermittent and uncoordinated gaiting or jumping; backwards gaiting; flat body gaiting; turning on its axis; quiet; uncoordinated gaiting; still; jumps; head tremor; and *wetdog* shake (Supp Fig. S1 and Supp Table S2 for a more detailed description).

For each of those behaviours we measured the average time spent and the number of events (Supp Fig. S1) during 5-MeO-DMT or saline ICV injection. We found that 5-MeO-DMT ICV sessions (all doses together) led to a significant increase of mean time spent and the number of events of the behaviour ‘intermittent uncoordinated gaiting or jumping’, ‘uncoordinated gaiting’ and ‘still’ (Supp Fig. S1). To better understand and visualise the results we separated the behaviours in two groups: ‘still’ and ‘altered behaviours’ (a combination of behaviours 1 to 6), and evaluated the contribution of each dose to those two classes of behaviours (Fig. 1C, ANOVA with Tukey–Kramer post hoc test). We found that 5-MeO-DMT *vs* saline is significantly different for both behaviours (ANOVA, #*p* < 0.05, ‘Still’: Mean time: F(3,20) = 7.6747, Number of events: F(3,20) = 5.3164. ‘Altered behaviours’: Mean time spent: F(3,20) = 3.2362, Number of events: F(3,20) = 3.8690). More specifically, both the mean time spent and the number of events of ‘still’ are significantly decreased for every dose compared to saline. Conversely, there was an increase of the mean time and number of events of ‘altered behaviours’ for the dose ICV 200 ug compared to saline (Fig. 1C, Tukey–Kramer post hoc test, *p < 0.05; **p < 0.01).

### Electrophysiological effects of 5-MeO-DMT

We next evaluated the impact of 5-MeO-DMT on the spectral profile of hippocampal LFPs. As the drug significantly affected locomotion (Fig. 1B) and speed is known to influence LFPs, we employed a generalised linear model (GLM) to assess the effect of 5-MeO-DMT administration on delta (1–4 Hz) and theta (5–12 Hz) power while accounting for speed (Fig. 2, see Methods for details). To achieve this, we first used the GLM to predict the relative delta or theta power (compared to the baseline session) while controlling for speed. Our analysis showed that there was a significant and dose-dependent impact of 5-MeO-DMT on delta power in the hippocampus following IP injections (Fig. 2C–E, 5-MeO-DMT vs. saline: *p* = 0.001; Dose: *p* < 0.001). Interestingly, we did not observe the same effect for ICV injections. In contrast, we observed a significant decrease of theta power following the administration of 5-MeO-DMT using both IP (Fig. 2I–K) and ICV (Fig. 2L–N) delivery, with a significant dose-dependent effect specifically for ICV injections (5-MeO-DMT vs. saline: *p* < 0.001; Dose: *p* = 0.001). In summary, our findings indicate that 5-MeO-DMT administration leads to a decrease in theta power in the hippocampus for equivalent locomotion speeds, as well as an increase in delta power specifically following IP injections.

**Figure 3:**
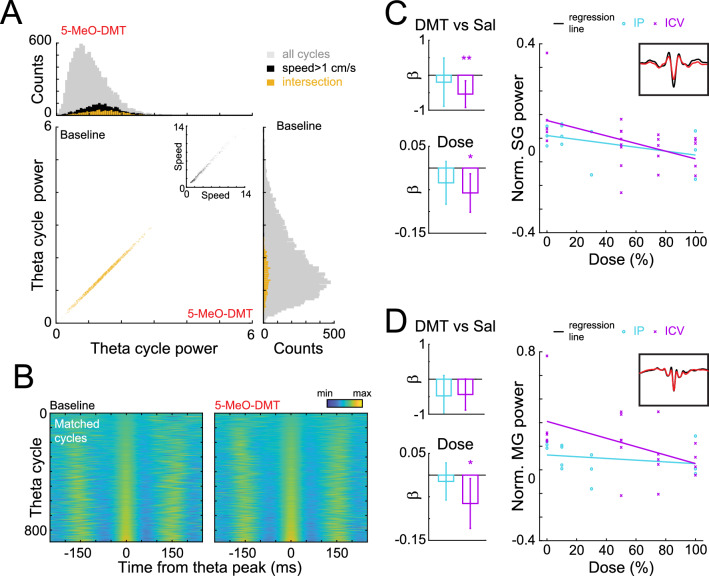
Slow- and mid-gamma power changes in the hippocampus after 5-MeO-DMT injection. (**A**) Example of theta cycle matching procedure. Each theta cycle occurring after the injection was matched to a theta cycle in baseline with a similar theta envelope and animal speed. (**B**) Representative recording day for matched theta cycles between baseline and post-injection conditions. Each row in the baseline panel corresponds to a distinct theta cycle, which is paired with a cycle of matched amplitude in the same row of the 5-MeO-DMT panel. The theta-filtered traces are depicted using pseudocolors and aligned to the corresponding theta peaks, providing a visual representation of the similarities between the two conditions. (**C**) The average slow-gamma envelope was then computed for each cycle in B. Bar plots show the coefficients of a GLM used to estimate slow-gamma power from the type of injection (5-MeO-DMT vs. saline; top) or from different doses of 5-MeO-DMT (bottom) as a variable. In the latter case, doses were normalised from 0 (saline) to 100% (maximal dose) (left). GLMs for IP and ICV were run separately. Error bars denote 95% confidence interval and inset shows a representative slow-gamma triggered average. (**D**) Similar to C, but for mid-gamma power.

Hippocampal theta oscillations are known to nest different gamma oscillations that reflect distinct circuits and pathways (for a review see^36^. In alignment with previous studies^47,49–53^, we divided the gamma range in two components: slow (20–50 Hz) and mid (50–100 Hz). To assess the impact of 5-MeO-DMT administration on these two gamma rhythms, we conducted a matching analysis that controlled for changes in speed and theta power (Fig. 3A,B; see Methods). This analysis revealed a significant dose–response relationship between 5-MeO-DMT ICV injection and slow gamma power (Fig. 3C, 5-MeO-DMT vs. saline: *p* = 0.008; Dose: *p* = 0.013). Specifically, slow gamma amplitude tended to be lower when nested in theta cycles during the post-injection epoch, compared to the baseline with matched power and speed. We observed a similar dose–response effect for mid gamma power (Fig. 3D, 5-MeO-DMT vs. saline: *p* = 0.053; Dose: *p* = 0.027). Notably, our results indicate that IP injections had no discernible effect on either slow or mid gamma power.

**Figure 4:**
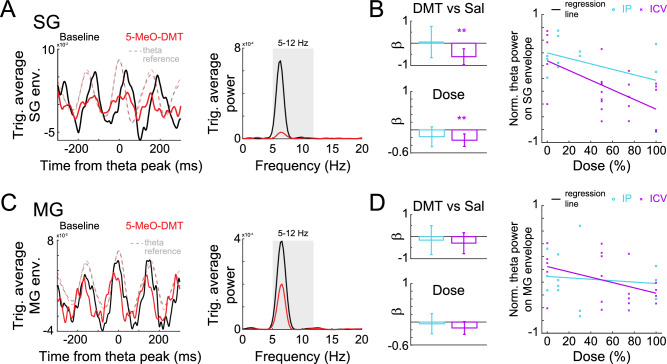
Hippocampal theta-nested slow- and mid-gamma oscillations after 5-MeO-DMT. (**A**). (Left) Average of slow-gamma envelope, triggered by theta peaks (dashed lines) after 5-MeO-DMT injection (red) and during baseline (black). (Right) Power spectrum of the triggered average on the left. Modulation was estimated by the average power in theta frequency band (shaded area). Theta cycles from baseline were matched in both mean theta amplitude (average envelope) and animal speed to account for the influence of those variables into gamma coupling. (**B**). Coefficients of GLM of theta-SG modulation, estimated using the injected drug (top) and taking the dose into account (bottom, right) for IP (light blue) and ICV (magenta) injections. GLMs for IP and ICV were computed separately. (**C**,**D**). Same as A-B, but for mid-gamma.

We proceeded to investigate the impact of 5-MeO-DMT on gamma amplitude modulation by the theta rhythm in the HP. To address the decrease in theta power caused by the drug we integrated theta amplitude into our matching analysis, enabling us to examine any additional effects on modulation beyond this reduction alone. Specifically, we selected individual theta cycles from pre- and post-injection epochs with matching amplitude and speed. We then calculated the average of the envelope of each gamma band, aligned to those theta peaks (see Fig. 4A,C). To evaluate theta modulation, we computed the power spectra of the triggered average envelopes (see methods) and quantified the power within the theta range. A higher concentration of power within the theta range of the triggered envelopes indicates a stronger modulation between the theta phase and the gamma amplitude.

Following this analysis, we found that 5-MeO-DMT administration resulted in a significant reduction in the theta modulation of slow gamma oscillations specifically after ICV administration (5-MeO-DMT vs. saline: *p* = 0.002; Dose: *p* = 0.003). Furthermore, we found no effect on the theta modulation of mid gamma oscillations in the HP.

**Figure 5:**
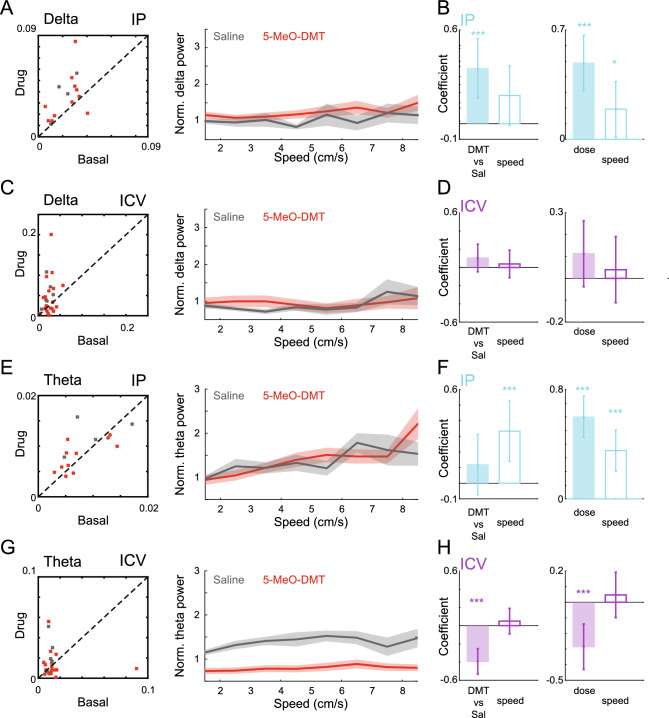
Changes in mPFC spectral power after IP and ICV 5-MeO-DMT dosing. (**A**) (Left) Mean delta power acquired from the bootstrap analysis during baseline and after dosing. Each cross corresponds to one experimental session (5-MeO-DMT: red; Saline: grey) after dosing. (Right) Mean normalised delta power of saline and drug conditions. (**B**) GLM delta power for IP dosing. (**C** and **D**) Similar to A and B, for theta band. (**E**–**H**) Similar to A–D, for ICV dosing. Notice the decrease in theta normalised power after 5-MeO-DMT in comparison to saline dosing.

Next, we investigated the impact of 5-MeO-DMT on prefrontal cortex delta and theta power (Fig. 5) using the same analysis framework for the HP channels (Fig. 2). Our findings indicate that IP injections of 5-MeO-DMT led to a dose-dependent increase in delta (5-MeO-DMT vs. saline: *p* < 0.001; Dose: *p* < 0.001) and theta power (5-MeO-DMT vs. saline: *p* = 0.002; Dose: *p* < 0.001), while ICV administration resulted in a significant dose-dependent decrease of theta power (5-MeO-DMT vs. saline: *p* < 0.001; Dose: *p* < 0.001). These opposite effects suggest that the route of administration plays a critical role in the impact of 5-MeO-DMT on prefrontal cortex delta power. Further, we observed no significant effect for the theta range with IP injection, whereas ICV administration led to a dose-dependent decrease of theta power, consistent with the HP results.

To analyse the spectral patterns of 1-s windows of combined hippocampal and cortical activity, we used a manifold approach called *state map*^38^. This approach generates a two-dimensional representation of LFPs by calculating the ratio of two frequency bands: (1) delta over theta plus delta, and (2) from delta to beta over delta to gamma (see Methods for details). The state map method was primarily designed to identify three main clusters of the sleep–wake cycle: WK, SWS, and REM. In summary, high amplitude theta power, i.e., low ratio (1), distinguishes WK and REM from SWS, whereas relative gamma power on theta oscillations separates WK from REM (WK theta cycles exhibit higher gamma power compared to REM cycles, captured by ratio (2).

**Figure 6:**
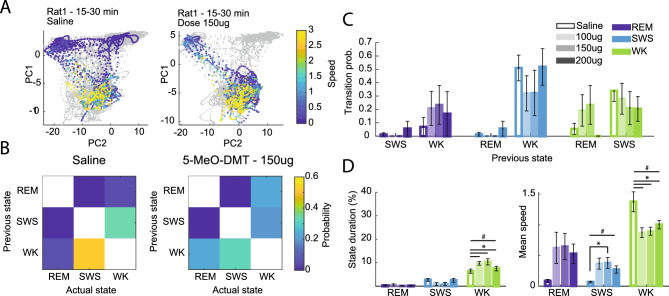
State map spectral changes resemble the sleep–wake cycle after ICV 5-MeO-DMT dosing. (**A**). Representative examples of state maps of 5-MeO-DMT experiments. Grey trace represents the baseline period. (**B**) Transition matrix showing the probability of change between each pair of states for saline (left panel) and 5-MeO-DMT 150 ug experiments. (**C**) Transition probability to REM (dark blue), SWS (light blue) and WK (green), given the specified previous state (x-axis). Empty bars for saline and filled bars for 5-MeO-DMT at doses 100 ug, 150 ug and 200 ug (from lighter to darker colours). (**D**). Percentage of state duration (left panel) and mean speed (right panel) of each sleep-waking state for saline and different doses of 5-MeO-DMT experiments (ANOVA, #*p* < 0.05 (WK: State duration: F(3,27) = 3,638); SWS: Mean speed: F(3,320) = 3,0864; WK: Mean speed: F(3,1560) = 4,7072), post hoc test *p < 0.05, SEM; n = episodes of sleep or wake).

We first used the state maps of the baseline session of each animal to define the three main sleep-wake clusters (see Supp Fig. S3,S4 for examples). Then, we projected the activity of post-injection windows onto that map (Fig. 6A and Supp Fig. S2A). Consistent with our previous results, this analysis revealed that animals exhibited an altered spectral profile after 5-MeO-DMT dosing. Once we had the state map for pre- and post-dosing periods for saline and drug experiments, we calculated the transition between each pair of states (Figs. 6B). We found that saline and 5-MeO-DMT groups had similar transition probabilities (Figs. 6C for ICV, Supp Fig. S2B for IP). Notwithstanding, transitions between WK-like and REM-like states tended to increase for 5-MeO-DMT ICV experiments—especially for the doses 100 ug and 150 ug (Fig. 6B; see the increase in the mean of transition probability for the WK purple bars in Fig. 6C).

Next, we computed the duration and animal speed (locomotion) for each sleep or wake episode. We found a significant increase in the time spent in WK state after 5-MeO-DMT ICV dosing, while it did not change for REM-like or SWS-like states compared to saline (Fig. 6D, left panel). Conversely, we found that mean speed across episodes was increased during REM-like and SWS-like states, while it decreased during WK after 5-MeO-DMT IP (Fig. 6D, right panel). Animals did not spend more time in a sleep state; they rather presented a greater speed during that sleep-like state. In that sense, we could say that there was a significant change in the state map profile that was not accompanied by the behavioural state. In other words, 5-MeO-DMT administration pushed the hippocampal activity closer to the states observed during baseline sleep, as indicated by post-5-MeO-DMT periods invading the SWS and REM clusters in the state map. Note in the representative examples the abnormal transitions (Fig. 6B, left panel) and high speed cluster displacement from WK, an effect not seen for saline (Fig. 6D right).

We found similar state map changes for the 5-MeO-DMT IP dosing, even though the transition probability between sleep-like and wake-like clusters did not change significantly (Supp Fig. S2B), Likewise in ICV experiments, in the IP experiments there was an increase in time spent in WK and a decrease in the WK mean speed across episodes (Supp Fig. S2C). The opposite occurred for the SWS-like state, for animals spent more time and at higher speed within the SWS-like state after 5-MeO-DMT IP dosing (Supp Fig. S2C). In other words, the rats presented a SWS-like state even though they were actively moving across the open field. A similar yet non-significant trend occurred during the REM-like state; the REM-like regions at the dose of 10 mg had higher speed compared to saline (Supp Fig. S2C, right panel). In this case, animals not just presented a displacement of higher speeds cluster, but also such displacement occurred during a longer period and it seems to be towards the SWS-like cluster (or even to REM-like). Note that effect in the examples shown in Supp Fig. S2A. Rats 3–5 presented a high-speed cluster closer to SWS-like compared to Rat5 during saline (Supp Fig. S2A, right-bottom panel).

## Discussion

This study provided the first behavioural and electrophysiological quantification of speed-controlled LFP simultaneously recorded from the HP and the mPFC of freely behaving rats subjected to an acute psychedelic experience induced by 5-MeO-DMT at various doses.

### Behavioural changes induced by 5-MeO-DMT

Animals treated with 5-MeO-DMT exhibited a variety of altered behaviours (Fig. 1, Supp Fig S1), some of which are well documented stereotyped behaviours, while others were not previously reported in the literature. This may be attributed to the fact that most studies assess behavioural effects of drugs on specific tasks, rather than on spontaneous activity. Our findings indicate that the most significant changes in behaviour were related to ‘uncoordinated gaiting’ and ‘stillness’.

The ‘Uncoordinated gaiting’ behaviour observed in our study may be related to the ‘forepaw treating’ associated with serotonergic syndrome^54^. Spanos et al. showed a dose-dependent increase in “forepaw treating” and ‘low body posture’, but not in ‘head weaving’ after MDMA injection in rats^55^. Conversely, we did not observe a significant change in the behaviour equivalent to ‘flat body gaiting’ or ‘low body posture’.

In accordance with the appearance of altered behaviours, we found that animals exhibited a lower level of stillness (both in duration and frequency) following administration of 5-MeO-DMT at all doses compared to when they were given saline (Fig. 1B and C).

Although the remaining quantified behaviours did not change individually, they did show a general increase in prevalence following administration of 5-MeO-DMT. When grouping behaviours 1 to 6 as “altered behaviours,” we observed a significant increase in both the duration and frequency of these behaviours (Fig. 1C). This effect appeared to be dose-dependent, with a stronger impact observed at higher doses of the drug. While the behaviours of ‘backward gaiting’, ‘head tremor’ and *‘wetdog* shake’ did not show significant changes when considered individually, we observed an increase in their prevalence when analysed together, consistent with observations reported in mice treated with d-LSD^13^. *Wetdog* shake, also known as head-twitch response (HTR), is a rapid and sudden side-to-side shaking of the head/neck that is a well-known marker of the agonistic effect of the serotonergic system^56,57^. This behaviour is highly rhythmic and produces a wave-like oscillation around 90 Hz in mice^57^. In this study, we observed the occurrence of ‘*wetdog* shake’ in rats treated with 5-MeO-DMT (Supp Fig. S1B), which is consistent with evidence indicating that HTR is increased by other psychedelics in mice, such as DOI, DOM, and N,N-DMT^58^. However, we did not observe a significant change in the flat body gait behaviour (Supp Fig. S1), as previously reported for d-LSD^13^. Likewise, we found no significant increase in the duration of ‘turning around its axis’, a behaviour that has not been well described in the current literature on psychedelics, although 5-MeO-DMT tended to increase the number of events.

The differences observed between ICV and IP dosing may be related to the availability of 5-HT agonists, peripheral action, and the amount of drug that reaches the central nervous system. Our IP findings contrast with a similar study by Halberstadt et al.^12^, which found no initial increase in locomotion, but reported a later hyperactivity^59^. However, it is important to note that lower doses were used in their study, potentially leading to a subliminal or delayed effect on locomotion. The increase in locomotion we observed in the present study could be an effect of higher doses and more direct route of administration in the ICV experiments.

### LFP power changes induced by 5-MeO-DMT

We found a significant decrease of hippocampal theta power in a dose–response manner that could not be explained by changes in animal speed. This is in accordance with the idea that the median raphe acts as a theta desynchronization nucleus^60–62^. Moreover, the power of theta-associated gamma oscillations and their coupling to theta phase were significantly reduced by 5-MeO-DMT. The administration of 5-MeO-DMT induced significant alterations in animal locomotion, which is an important factor to consider when interpreting the potential effects observed in electrophysiology, as speed can have a profound impact. It is worth noting that our study differs from previous investigations in that we took this confounding variable into consideration.

### Differences between IP versus ICV experiments

The differences between IP and ICV dosing are also noteworthy. In the hippocampus, the dose-dependent increase in delta power following IP injections was not observed for ICV injections, implying a possible peripheral mechanism. Classical psychedelics such as 5-MeO-DMT can also bind to serotonergic receptors of the peripheral nervous system (PNS) and this could affect the CNS quite differently from the ICV dosing. Serotonin is a major controller of the gut motility, including intrinsic reflex, epithelial secretions, and vasodilatation. Furthermore, its signalling takes part in vagal extrinsic and spinal afferent fibre activation, leading to pancreatic secretion, satiation, pain and discomfort, nausea, and vomiting^63^. The high availability of serotonin in the PNS provided by the IP dosing may have led to a peripheral increase in receptor activation, causing physiological alterations such as gastric discomfort. Although not life-threatening, those alterations could potentially change the subjective experience of the animal during the experience, as well as its neural correlates. A second non-exclusive hypothesis is that the peripheral binding of psychedelics causes fewer molecules to reach the CNS. Yet, these putative lower doses were still effective, as shown by the significant electrophysiological changes observed in the HP and mPFC for theta and gamma oscillations after IP injections of 5-MeO-DMT. Regarding theta-nested gamma oscillations, IP injections produced less dose-dependency than ICV injections, which suggests that IP injections either dampen or shift the dynamic range of the effective intracerebral doses within the HP or mPFC.

### Changes in sleep and waking states induced by 5-MeO-DMT

State maps based on LFP power ratios provide a consistent measure of state-dependent spectral features as animals alternate between sleep and waking^38^. Under drug-free conditions, state maps usually display very similar patterns across animals, despite inter-individual differences in behaviour and LFP signals. In the present study the state maps presented marked alterations after psychedelic dosing compared to saline, with some changes shared by the two groups investigated (Fig. 6 and Supp Fig. S2). More specifically, ICV 5-MeO-DMT dosing did not influence the duration of SWS-like episodes but rather increased the animals’ speed during this spectral state (Fig. 6C). This result indicates that the animals treated with ICV 5-MeO-DMT were behaviourally awake, but their HP seems to be in an SWS-like state. There was also an increase in the duration of WK episodes, although the animals’ speed decreased after ICV 5-MeO-DMT (Fig. 6C). Therefore, when animals had electrophysiological features of WK, they were more likely to be at a lower speed after ICV 5-MeO-DMT compared to saline.

The results obtained in the IP 5-MeO-DMT experiments were overall like those from the ICV 5-MeO-DMT experiments, with animals spending more time in the SWS-like and WK-like spectral regions (Supp Fig. S2C, left panel), and presenting higher speeds during the SWS-like state and lower speeds during the WK-like state, when compared to animals treated with saline (Supp Fig. S2C, right panel).

### Study limitations

One limitation of our study is that we conducted all experiments exclusively on male rats, aligning with the predominant use of male rodents in both behavioral (e.g.,^12–15,17^) and electrophysiological (e.g.,^19,23^) psychedelic research. Our primary aim was to confirm and build upon existing electrophysiological findings, leading us to maintain methodological consistency with previous research. Nonetheless, we recognize that this standard approach stems from the outdated belief that hormonal cycles in female rodents contribute to increased experimental variability, a notion recent literature has debunked^64–66^. Given this, we acknowledge that the exclusive use of male rats is a limitation of our study, and future research should consider including female subjects to address this gap and enhance generalizability.

Another important limitation is that we did not investigate the receptors involved in the effects described. 5‐MeO‐DMT is mainly a nonselective serotonergic agonist, with some binding to the serotonergic and noradrenergic transporters, as well as dopamine receptors^59,67^. Further studies are therefore needed to specify the receptor dependence underlying the effect.

## Conclusions

In this study we have given special attention to the effects observed in the hippocampus. Focusing on the hippocampus in psychedelic studies presents a compelling avenue due to its central role in several critical cognitive functions. The hippocampus emerges as a main hub of neural plasticity, a core attribute that psychedelics notably affect^4,68–71^. Such plasticity is foundational for memory and learning—domains where the hippocampus is crucial. Investigating the influence of psychedelics on hippocampal neuroplasticity allows researchers to delve into novel perspectives on how these compounds might bolster cognitive adaptability and offer therapeutic avenues for diverse psychiatric disorders. Furthermore, the complex electrophysiological dynamics of the hippocampus have been thoroughly investigated in freely moving rodents during both waking and sleep^36,41,52,72–76^. This depth of understanding not only streamlines the interpretation of alterations induced by psychedelics in these neural signals but also enhances our capability to conduct comprehensive monitoring of the sleep–wake cycle through hippocampal signal analysis^38^.

Overall, the results presented here strengthen the notion that psychedelics induce significant changes in the sleep–wake cycle, possibly leading to sleep-like waking states of hippocampal LFP oscillations^28^. They also resonate with early studies of the effects of d-LSD in humans. Muzio et al.^77^ found that low doses (6–40 ug) caused a prolongation of the first and second REM sleep episodes, and a shortening of subsequent episodes, with brief REM sleep episodes interrupting SWS. Another study showed a decrease in latency between the bouts of REM sleep after dosing, with a decrease in theta activity^78^. Altogether, the available evidence suggests that the psychedelic experience precludes sleep at the behavioural level yet allows the brain to continue cycling across waking-like and sleep-like electrophysiological states. We speculate that this might underlie the alternation between extroverted and introverted periods during the psychedelic experience, in line with the increased repertoire of metastable states induced by psilocybin^79^.

It is important however to consider that the LFP analysis of the WK-like, SWS-like and REM-like spectral regions of state maps does not comprise the behavioural parameters that characterise sleep–wake states, such as immobility or muscle atonia. Furthermore, the SWS-like or REM-like spectral regions are not equivalent to the SWS or REM spectral regions, but rather reflect proportional spectral features that point to similar frequency patterns across sleep states. In other words, the altered states induced by 5-MeO-DMT spectrally resemble the regular states of the wake-sleep cycle, but also differ from them in many respects. Further studies shall clarify whether the sleep-like electrophysiological features induced by psychedelics are indeed related to the dream-like psychological features of the psychedelic experience.

### Supplementary Information

Supplementary Information.

## Data Availability

The data will be made available by Sidarta Ribeiro upon reasonable request.
