# Author Correction: 5-MeO-DMT induces sleep-like LFP spectral signatures in the hippocampus and prefrontal cortex of awake rats

**DOI:** 10.1038/s41598-024-66730-6

**Published:** 2024-07-10

**Authors:** Annie C. Souza, Bryan C. Souza, Arthur França, Marzieh Moradi, Nicholy C. Souza, Katarina E. Leão, Adriano B. L. Tort, Richardson N. Leão, Vítor Lopes-dos-Santos, Sidarta Ribeiro

**Affiliations:** 1 Brain Institute, Federal University of Rio Grande do Norte, Natal, Brazil; 2 Department of Psychology, Florida State University, Tallahassee, USA; 3 Donders Institute for Brain, Cognition and Behaviour, Radboud University, Nijmegen, The Netherlands; 4 Netherlands Institute for Neuroscience, Amsterdam, The Netherlands; 5 Department of Neuroscience and Behavioural Sciences, School of Medicine, University of São Paulo, Ribeirão Preto, Brazil; 6 Medical Research Council Brain Network Dynamics Unit, Nuffield Department of Clinical Neurosciences, University of Oxford, Oxford, UK; 7 Center for Strategic Studies, Oswaldo Cruz Foundation (FIOCRUZ), Rio de Janeiro, Brazil

Correction to: *Scientific Reports* 10.1038/s41598-024-61474-9, published online 17 May 2024.

The original version of this Article contained an error in Affiliation 6, which was incorrectly given as ‘Brain Network Dynamics Unit, University of Oxford, Oxford, UK.’ The correct affiliation is listed below:

Medical Research Council Brain Network Dynamics Unit, Nuffield Department of Clinical Neurosciences, University of Oxford, Oxford, UK.

The original Article has been corrected.

