# Supplementary material for: 5-MeO-DMT induces sleep-like LFP spectral signatures in the hippocampus and prefrontal cortex of awake rats
Source: Sci Rep. 2024 May 17;14:11281. doi: 10.1038/s41598-024-61474-9 (PMC11101617; doi:10.1038/s41598-024-61474-9)
Supplement: Supplementary file 1 — Supplementary Information. [file 41598_2024_61474_MOESM1_ESM.pdf]

SUPPLEMENTARY MATERIAL

Supplementary Table S1: Number of animals in each experimental group.

Number of sessions	Saline	5-MeO-DMT low dose	5-MeO-DMT mid dose	5-MeO-DMT high dose
IP, HP	9	4	3	4
IP, PF	4	4	3	4
ICV, HP	7	10	8	8
ICV, PF	5	8	6	6

Supplementary Table S2: Description of the behavioural analysis for the first 30 min after 5-MeO-DMT (ICV) or saline dosing.

	**Behaviour**	**Description**
**1**	Intermittent and uncoordinated gaiting or jumping	The animal displayed an intermittent behaviour of walking and stopping within short periods of interval (~10 s). While walking its paws presented an uncoordinated rhythm, and/or it moved by making sudden jumps forward.
**2**	Backward gaiting	The animal walked backwards with short/small or long steps.
**3**	Flat body gaiting	Animal displayed gaiting with lower posture, with his body (ventral core) collapsed to the floor.
**4**	Turning on its axis	The animal turned circularly with its head toward its tail basis, or its forepaws treading were making it turn to the side without moving its back paws.

**5**	Quiet	Periods when the animal was quieter than active awake, but not completely still.
**6**	Uncoordinated gaiting	Animal walked with its paws in an uncoordinated rhythm.
**7**	Still	Animal was completely still, very quiet, with no movements.
**8**	Jumps	Animal suddenly jumped forward.
**9**	Head tremor	The animal presented a head-tremor that was different from the *wetdog* shake, in a slower rhythm, as if it was biting its teeth.
**10**	*Wetdog* shake	The animal presented a very sudden head-twist from side to side, rapid with great lateral amplitude.

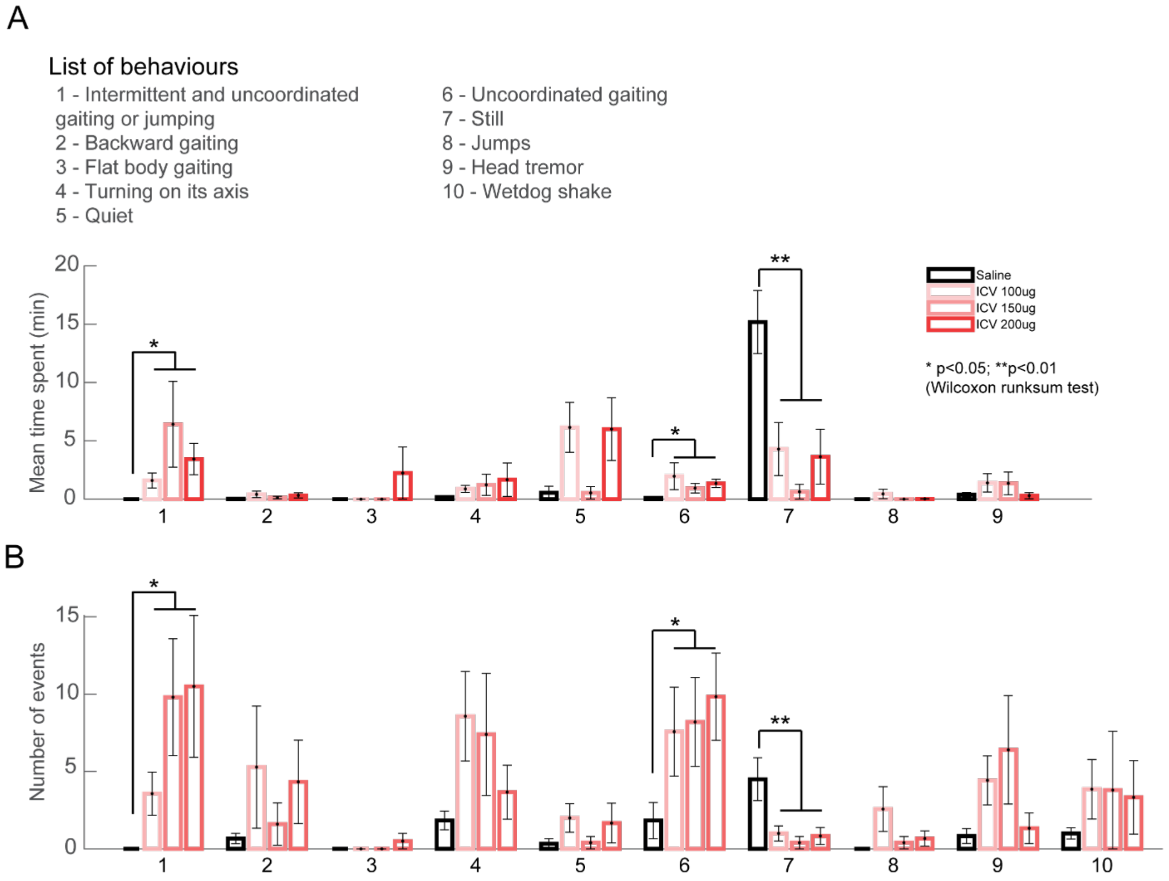

Supplementary Figure S1: **Behavioural changes after ICV 5-MeO-DMT dosing**. **(A)** Mean time spent in each behaviour (1 to 9) for ICV 5-MeO-DMT experiments for all ICV doses (100 ug, 150 ug and 200 ug). **(B)** The number of episodes of each behaviour (1 to 10) for ICV 5-MeO-DMT experiments, across all doses (100 ug, 150 ug and 200 ug). *p<0.05, **p<0.01, Wilcoxon rank-sum test for all conditions drug *versus* saline.

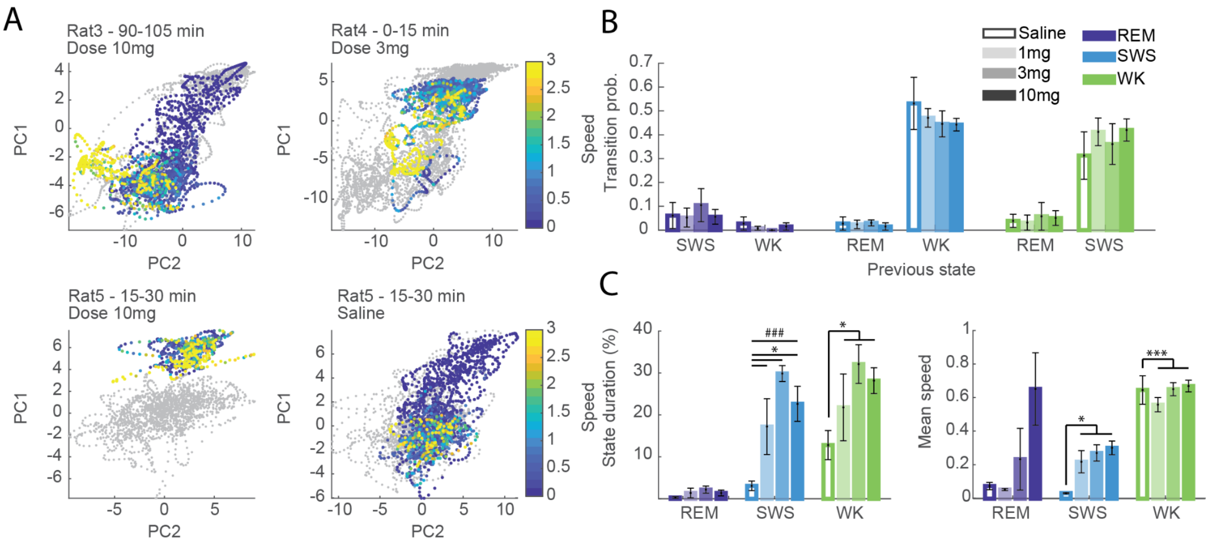

Supplementary Figure S2: State map changes after IP 5-MeO-DMT dosing. (A). Representative examples of state maps of 5-MeO-DMT experiments. Grey trace represents the baseline period; black refers to the post-dosing period and red to post-dosing periods of animal's speed >1 cm/s. Analyses performed for the post-dosing period of 30min (and up to 3 h 30 min, for some animals). **(B)** Transition probability to REM (dark blue), SWS (light blue) and WK (green), given the specified previous state (x-axis). Empty bars for saline and filled bars for 5-MeO-DMT at doses 1 mg, 3 mg, and 10 mg (from lighter to darker colours). **C.** Percentage of state duration (left panel) and mean speed (right panel) of each sleep-waking state for saline and different doses of 5-MeO-DMT experiments (State duration: ANOVA, ###p<0.001 (SWS: State duration: F(3,13)=10,3419), post hoc test, *<0.05, SEM; Mean speed: Wilcoxon rank-sum test, *p<0.05 ***p<0.001, SEM; n= episode of sleep or waking).

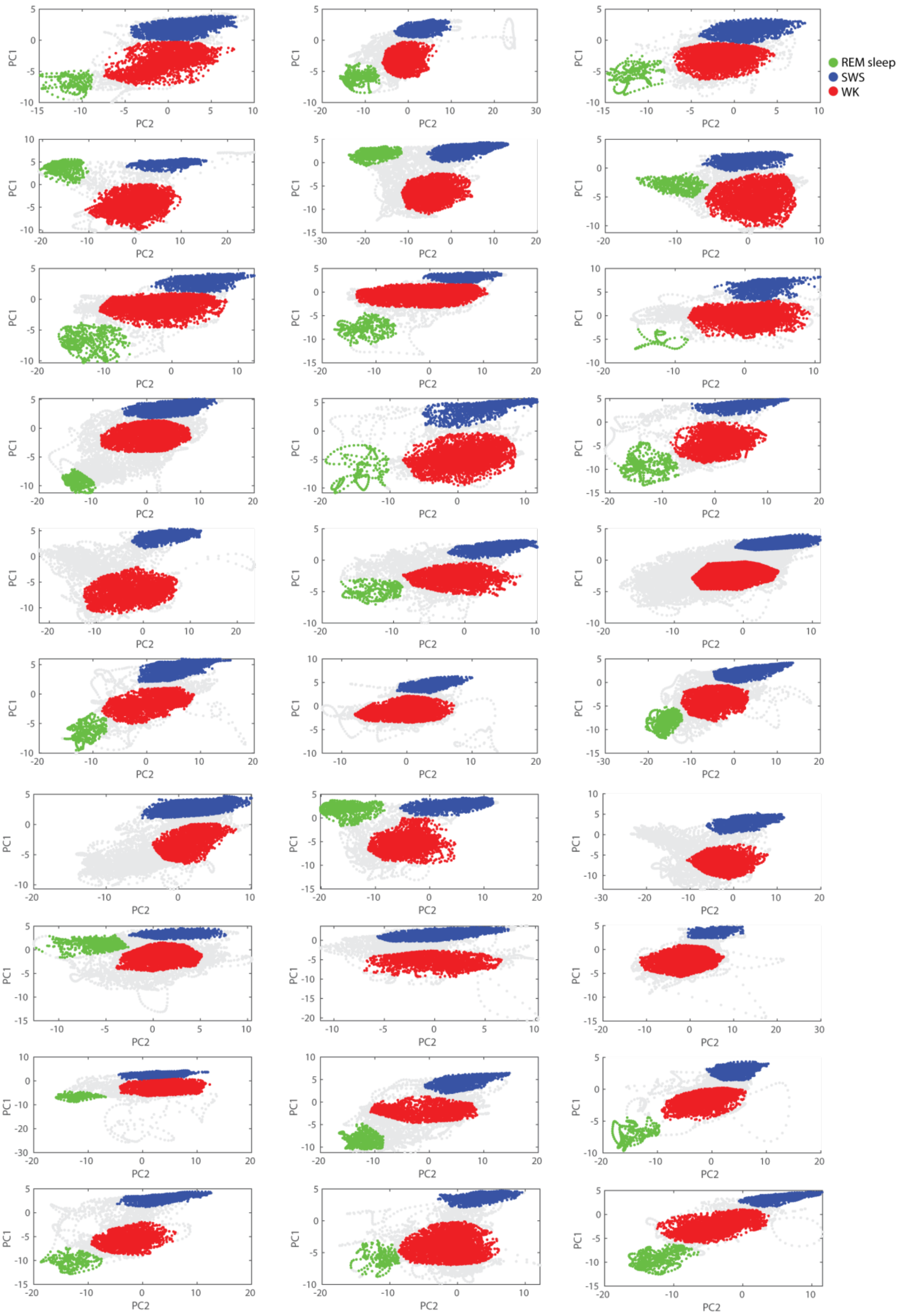

Supplementary Figure S3: Manual annotations of sleep states from the 2D spectral maps for 5-MeO-DMT ICV experiments. Each map is delimited for the baseline session and then the experimental period is assigned based on the baseline map previously defined. The assignments of waking periods were guided by the occurrence of high animal's speed. Both baseline and drug periods are shown in this figure. Each dot corresponds to 1 s period. WK, SWS and REM sleep periods are shown in red, blue, and green, respectively.

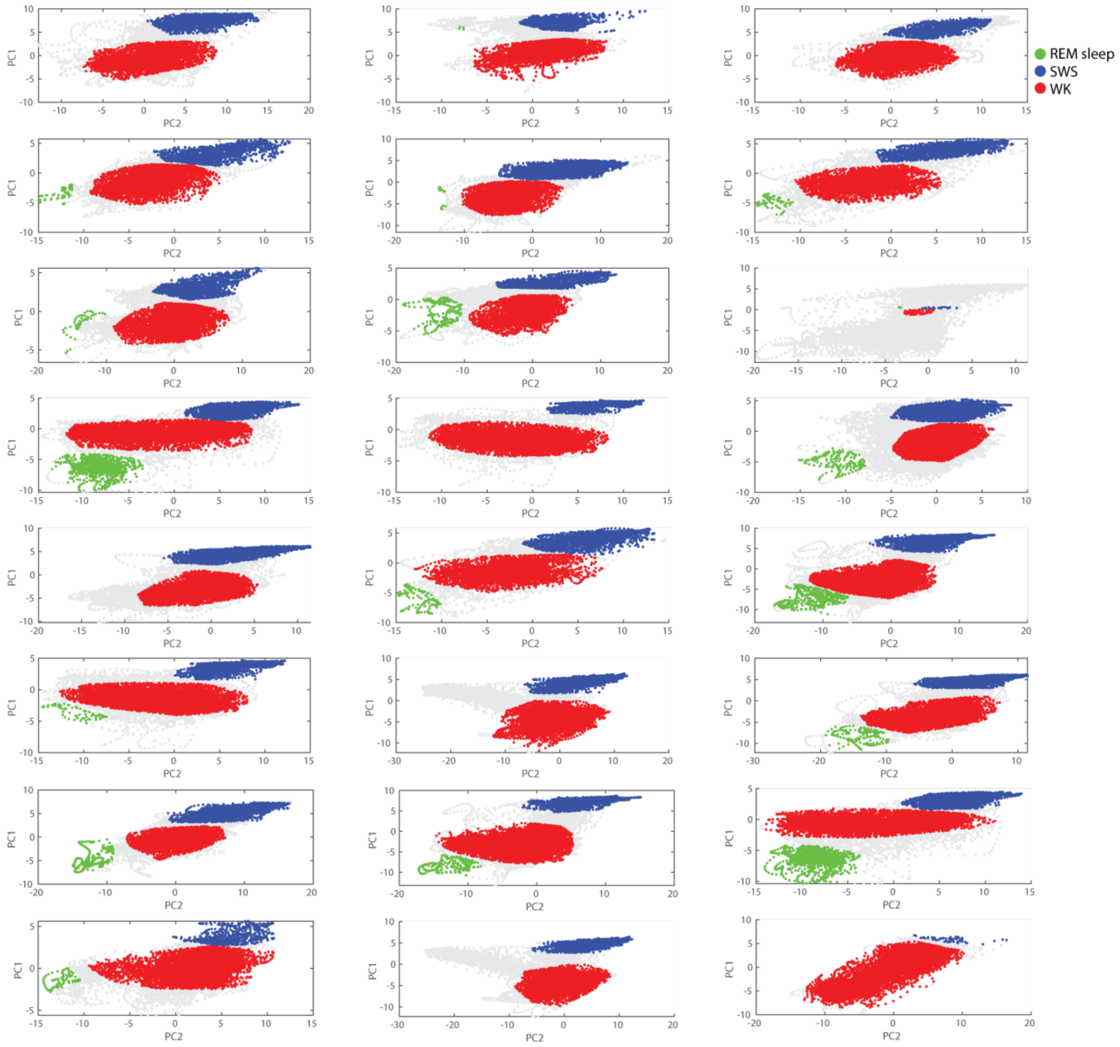

Supplementary Figure S4: Manual annotations of sleep states from the 2D spectral maps for 5-MeO-DMT IP experiments. Each map is delimited for the baseline session and then the experimental period is assigned based on the baseline map previously defined. The assignments of waking periods were guided by the occurrence of high animal's speed. Both baseline and drug periods are shown in this figure. Each dot corresponds to 1 s period. WK, SWS and REM sleep periods are shown in red, blue, and green, respectively.
